# Impact of Loneliness on Functioning in Lung Cancer Patients

**DOI:** 10.3390/ijerph192315793

**Published:** 2022-11-27

**Authors:** Jacek Polański, Weronika Misiąg, Mariusz Chabowski

**Affiliations:** 1 Department of Internal Medicine, Occupational Diseases, Hypertension and Clinical Oncology, Wroclaw Medical University, 50-556 Wroclaw, Poland; 2 Student Research Club No. 180, Faculty of Medicine, Wroclaw Medical University, 50-367 Wroclaw, Poland; 3 Department of Nursing and Obstetrics, Division of Anesthesiological and Surgical Nursing, Faculty of Health Science, Wroclaw Medical University, 51-618 Wroclaw, Poland; 4 Department of Surgery, 4th Military Teaching Hospital, 50-981 Wroclaw, Poland

**Keywords:** lung cancer, loneliness, QoL, malnutrition, functioning

## Abstract

Lung cancer is the leading cause of cancer death and carries a greater degree of stigma. Lung cancer stigma contributes to social isolation and increases loneliness, which has an impact on quality of life, increases depressive symptoms and hence affects all aspects of functioning. Functioning is assessed in five dimensions (physical, psychological, cognitive, social and life roles). The aim of the study was to assess the impact of loneliness on the functioning, nutritional status and quality of life of patients with lung cancer. METHODS. The survey study was conducted among 310 lung cancer patients. The patients were asked to complete the Mini-MAC, HADS-M, MNA, EORTC QoL, AIS and VAS questionnaires. RESULTS. In total, 136 patients were lonely and 174 were married or in a relationship. Lonely patients had significant difficulty accepting their illness and demonstrated higher levels of depression. The factors most strongly associated with loneliness were being unemployed, age 61 or over and a less score in the constructive coping strategies. CONCLUSIONS. Loneliness is a significant factor affecting the functioning of patients with lung cancer. It increases the risk of anxiety and depression, reduces levels of illness acceptance, reduces levels of constructive coping and increases the risk of malnutrition.

## 1. Introduction

Lung cancer accounts for 13% (1.6 million) of new cancer cases worldwide (12.7 million). It is the most commonly diagnosed cancer in men and the leading cause of cancer death (1.4 million, 18% of cancer deaths worldwide) [1]. There are many risk factors for lung cancer and a number of clinical conditions that are associated with an increased incidence of the condition. These include idiopathic pulmonary fibrosis, systemic sclerosis, dermatomyositis, ascites and asbestosis [2,3]. With advances in cancer detection and treatment, the number of cancer survivors has been increasing. The improved cancer survival mandates attention to the functioning of patients, their quality of life (QoL), including their mental and physical health, and their experience of loneliness [4]. Functioning is assessed in five dimensions (physical, psychological, cognitive, social and life roles). Functioning in patients with lung cancer is affected as follows: severity of symptoms, increasing disability, inability to fully fulfill family and social roles, sleep and respiratory problems, fatigue related to the disease and burdensome treatment [5]. Despite significant advances in the detection and treatment of lung cancer, the prognosis is often poor [6]. This is due to the severity of clinical symptoms and intensive treatment regimens [7], which significantly affect the mental health and QoL of patients and increase their loneliness [8,9,10]. In addition, patients with lung cancer belong to the group of patients with a relatively high frequency of malnutrition. Low nutritional status also affects the QoL, physical performance and survival rate [11].

Loneliness is a painful experience for patients. We can distinguish loneliness in family and social relationships and loneliness in relationships with healthcare professionals [12]. Loneliness is a very important psychosocial factor for cancer patients. It has a negative impact on their QoL [13,14], precedes sleep difficulties [15], physical [16] and functional inactivity [17], increases their depressive symptoms [18,19] and thus affects all aspects of their functioning [8]. Lung cancer carries a greater degree of stigma than other types of cancer [20]. The stigma results from the fact that lung cancer patients are assumed to be smokers, regardless of whether they have a history of smoking [21,22]. This causes them to feel shame and guilt [21,23]. As a result, patients delay visiting a doctor for their worrying symptoms, which delays the diagnosis and has a negative impact on the prognosis. The stigma contributes to the social isolation of patients, increasing their loneliness.

The aim of the study was to assess the impact of loneliness on the functioning, nutritional status and quality of life of patients with lung cancer.

## 2. Materials and Methods

The prospective, cross-sectional, survey study was conducted among 310 patients treated for lung cancer at the Lower Silesian Oncology Centre. The patients were aged between 25 and 87 years (median 64 years). The patients were asked to complete the Mini-MAC, HADS-M, MNA (Mini-Nutritional Assessment) and EORTC QoL questionnaires as well as the Acceptance of Illness Scale (AIS) and the Visual Analogue Scale (VAS), which measures pain intensity. Patients also completed a questionnaire on sociodemographic data such as follows: level of education, domicile, employment status (working professionally or unemployed, on retirement, on benefits or disability pensions, state of relationship (lonely, married or in a relationship). The general and clinical characteristics of the patients studied are shown in Table 1 and Table 2.

The Mini-MAC scale is used to assess a cancer patient’s mental adjustment to their illness. It evaluates the following constructive and destructive cancer coping strategies: anxious preoccupation, fighting spirit, helplessness-hopelessness and positive redefinition. Each category includes 7 items and has a possible score range of 7–28. The higher the score, the stronger the behaviour typical of a given strategy. Scores on the Mini-MAC scale may also serve as a measure of health-related QoL [24].

We assessed the patients for depressive and anxiety symptoms using the HADS scale. The scale consists of 2 subscales, containing 7 items each. Each item is rated on a 4-point scale (0—no, not at all, 1—no, not much, 2—yes, sometimes, 3—yes, definitely). The tool yields separate scores for depression and anxiety. A score of 0–7 is classified as ‘normal’, a score of 8–10 is classified as a ‘borderline’ case and a score of 11–21 represents a ‘case’ of psychological morbidity [25,26].

We assessed the nutritional status using the Mini-Nutritional Assessment (MNA) questionnaire [27,28]. The questions are related to measures such as follows: BMI, mid-arm and calf circumference, weight loss in the past 3 months, lifestyle, dietary habits with the number of meals and fluid intake, mobility level and patient’s assessment of his/her health and nutritional status. In this questionnaire a maximum of 30 points can be achieved. Results below 17 points indicate about malnutrition, 17–23,5 about risk of malnutrition and a score of 24–30 points are defined as normal nutritional status.

We assessed the QoL of the patients using the EORTC QLQ-C30 questionnaire, which consists of 30 questions. It is used to assess physical, cognitive, emotional, social functioning and functioning in life roles. Moreover, the instrument has additional symptom scales (fatigue, nausea and vomiting, pain, dyspnoea, insomnia, loss of appetite, constipation, diarrhoea) and incorporates the perceived financial impact of the disease [29,30]. A score range in all sub-scales and individual items is from 0 to 100 points. A better function and a higher quality of life are represented by a higher score. However, a higher score achieved in symptom subscale, the greater severity of symptoms. All items and subscales are considered separately due to the impossibility of evaluating the total score. The scores are evaluated using normative data [31].

The Acceptance of Illness Scale (AIS) is used to assess the extent to which a patient has accepted their illness. It consists of 8 items with a 5-point scale concerning the negative consequences of illness. The higher the score, the higher the degree of illness acceptance. The scale can be used for any disease. In this study, we used it to assess the degree of lung cancer acceptance [32].

The Visual Analogue Scale (VAS) is used to measure pain intensity. It is a 10 cm line with the following two anchors: 0 = no pain and 10 = worst pain possible.

The study was conducted in accordance with the Declaration of Helsinki and approved by the Ethics Committee of Wroclaw Medical University No 729/2019.

Statistical methods. Continuous and discrete variables significantly differentiating lonely patients from those who were married or in a relationship were transformed into dichotomous variables. Threshold values were determined based on a ROC curve analysis. The proportions of patients in subgroups differing in marital status and functioning status, as assessed using the Mini-MAC, AIS and HADS questionnaires, results of independence tests and the values of odds ratios are shown in Table 3.

The reliability of the Polish version of the AIS is close to the original version in terms of consistency and stability (Cronbach’s alpha = 0.82). The Polish adaptation of the AIS scale developed by Juczyński was used in the study [33]. In earlier studies using the AIS scale, a relationship was found between a level of acceptance of the disease and the subjectively assessed quality of life. The MAC (Mental Adjustment to Cancer) scale was developed by M. Watson et al. The Polish language version prepared by Jurczyński has good psychometric properties [34]. The EORTC QLQ-C30 questionnaire is a questionnaire repeatedly used to assess the quality of life of patients with lung cancer. Excellent psychometric properties were used in this study group.

The sample size analysis was carried out based on one of the main primary objectives, which assumed at least 20% less a cancer patient’s mental adjustment to their illness in people living alone compared to people who are married or in a relationship. The minimum sample size needed to detect this difference assuming alpha = 5%, power = 80% and confidence level = 95% is 282 patients in total. Additionally, the risk of not completing the questionnaires correctly was assumed at the level of 10%. The final sample size is 310 participants. Sample size analysis was performed using the G*Power program [35].

For descriptive data, proportions for qualitative variables and means with SDs or medians with interquartile ranges (IQR) (25th–75th) for quantitative variables were used. The normality of distribution of variables was assessed using the Kolmogorov–Smirnov test and the Shapiro-Wilk goodness-of-fit test. Comparisons between lonely patients and those who were married or in a relationship were carried out using the Chi-square test or the z-test for two proportions for qualitative variables or the Student’s *t*-test or Mann–Whitney U-test for quantitative variables, as appropriate. Baseline factors associated with loneliness were analysed using univariate and multivariate logistic analysis. Variables yielding *p*-values of less than 0.2 in the univariate analysis were considered for inclusion in the multivariate analysis. The *p* < 0.2 level was adopted arbitrarily. The idea was not to overlook factors in multivariate analysis that in univariate analysis weakly correlate with the dependent variable, but in interaction with other independent variables may have a significant impact on the response of the model. The multivariate analysis (Table 4) did not include variables such as male sex (*p* = 0.564) and living in the city (*p* = 0.743), which were not significantly related to marital status (Table 1). The quality of the proposed logistic regression model was assessed using the Hosmer–Lemeshow test. All tests were two-sided at a significance level of 0.05. Statistical analysis was performed using the STATISTICA package, version 13.3 (TIBCO Software Inc., Palo Alto, CA, USA).

## 3. Results

Of the 310 patients studied, 136 (43.9%) were lonely and 174 declared that they were married or in a relationship. Among the surveyed people—patients who declared that they were married or in a relationship—there were no people who declared to feel lonely. Therefore, the groups were divided into subgroups of lonely and married or in a relationship. Men accounted for 55.1% (*n* = 75) of lonely patients and 58.6% (*n* = 102) of patients who were married or in a relationship. Only 10.3% of lonely patients were employed. For patients who were married or in a relationship, the percentage was 41.4%. Of the lonely patients, most had vocational education, followed by secondary education, then primary education and tertiary education. Of the patients who were married or in a relationship, the statistics were similar, except that a larger percentage were patients with tertiary education levels than primary education. The exact percentage results are shown in Table 1. The most common cancer stages were T2 and T4. More than 60% of lonely patients and more than 50% of patients who were married or in a relationship did not have distant metastases. The most common metastatic sites were the liver, adrenal gland, bone and brain.

The results are shown in Table 1. Lonely patients with lung cancer were older than patients married or in a relationship by an average of 6 years, had poorer education (*p* = 0.033) and were more likely to be unemployed (*p* < 0.001) compared with patients who were married or in a relationship. Lonely patients also tended to have a lower stage of primary cancer (*p* = 0.082).

General and clinical characteristics are presented in Table 2. In total, 39.7% of lonely patients and 44.8% of patients who were married or in a relationship had a history of smoking. Of the lonely patients, most had a moderate degree of airway obstruction, and a smaller group of respondents had a low degree of airway obstruction and a severe degree of airway obstruction. Comparable results are presented in the group of people married or living in a relationship.

In total, 50% of lonely patients and 48.3% of patients who were married or in a relationship had undergone surgery. The most commonly reported symptoms were chronic cough, dyspnoea, pain in the chest, haemoptysis, recurring infections, superior vena cava syndrome, arrhythmia and hoarseness, the frequency of which is shown off in Table 2.

Of the lonely patients studied, 35.3% had a satisfactory nutritional status, 44.9% were at risk of malnutrition and 19.9% were malnourished. Of the patients who were married or in a relationship, 44.3% had a satisfactory nutritional status, 37.9% were at risk of malnutrition and 17.8% were malnourished.

Lonely patients had more comorbidities and showed a higher degree of limitation in physical activity. Compared with patients who were married or in a relationship, lonely patients had lower scores on the fighting spirit and positive redefinition subscales of the Mini-MAC questionnaire, which measures the degree of mental adjustment to cancer.

Lonely patients were less likely to use the anxious preoccupation, fighting spirit and positive redefinition coping strategies compared with patients who were married or in a relationship. Thus, lonely patients showed lower levels of constructive coping (*p* < 0.001). Lonely patients had significant difficulty accepting their illness (*p* = 0.017) and demonstrated higher levels of depression (*p* = 0.034). A detailed analysis is presented in Table 3.

In our univariate analysis, the factors most strongly associated with being lonely were as follows: being unemployed, being age 61 or over and having a score of less than 41 for the use of constructive cancer coping strategies (Mini-MAC). Loneliness is considered an independent variable, and the dependent variable is defined as “Functioning in lung cancer”. As the factors analysed may have been correlated with one another, we performed multivariate analysis, the results of which are given in Table 4, which showed that the following were independent parameters associated with loneliness: being unemployed, having a cancer stage lower than T4 and having a score on the AIS of less than 21.

## 4. Discussion

Loneliness (being lonely) is a common experience of distressing social isolation and of one’s social needs not being met. It significantly affects daily functioning and has a negative impact on mental and physical health, which reduces patient satisfaction and decreases the Qol [25,36]. Simultaneously, depression is a strong predisposing factor for loneliness [36]. Factors influencing the occurrence of loneliness in our study included the age of 61 years and older, while the Deckx et al. meta-analysis showed no such relationship with age. However, it should be taken into account that in many studies evaluating loneliness in cancer patients, the mean age did not exceed 60 years [4]. The sense of loneliness is particularly acute among cancer patients. Studies have shown that loneliness in cancer patients is associated with both cancer-related factors, such as the stress associated with the diagnosis [37,38,39], the time since diagnosis, the type of cancer and the type and intensity of treatment, and non-cancer-related factors, such as the lack of psychological and social support and being unmarried (patients who have never been married, are widowed or divorced) [4]. Friedman et al. found that approximately 50% of the cancer patients studied felt lonely in situations relating to their illness [40]. Studies have also found that there is a relationship between social constraints and symptoms in cancer patients [41,42]. Lack of support, criticism and minimisation of symptoms may increase the severity of symptoms related to cancer or its treatment, such as fatigue, pain and sleep problems [43]. As a result, patients may use avoidance coping or even choose not to discuss their symptoms with anyone, including a doctor [44].

Loneliness is common among lung cancer patients. The illness carries a significant amount of stigma. Patients with lung cancer are often assumed to be smokers, even though the condition can also be diagnosed in non-smokers. Tobacco smoking is perceived as a poor life choice and those who make it are deemed to be responsible for their diagnosis [45,46]. This causes lung cancer patients to feel guilty. They feel angry, hurt and discriminated against. Moreover, they experience significantly higher levels of mental stress compared with patients with other types of cancer, which leads to social isolation [9,45,46,47,48,49]. As a result, patients with lung cancer are afraid of seeking help and support. Moreover, they may be afraid of consulting a doctor about their symptoms, which might delay diagnosis [44]. This may have very negative consequences, as rapid tumour growth and a lack of diagnosis significantly worsen the prognosis.

In addition, intensive therapy and exhausting symptoms caused by cancer or the side effects of its treatment make patients even less willing to take part in social interactions, which increases their loneliness. This cause-and-effect chain leads to lower illness acceptance and reduced willingness to maintain proper nutrition and engage in an appropriate amount of physical activity, which may entail malnutrition and depression. The coexistence of a mental disorder, malnutrition and loneliness has a negative impact on the prognosis of cancer patients and shortens their life expectancy. Studies have shown that loneliness is a significant factor predisposing to eating disorders, such as anorexia nervosa and is a significant predictor of malnutrition risk and malnutrition itself [50].

Cancer-related loneliness is associated with the high social expectations of patients. After a cancer diagnosis, patients may have idealised expectations of emotional support and their loved ones may not be able to meet them. When these expectations are not met, patients become distant from their family and friends and their loneliness increases. Cancer patients feel that they have to cope with their difficult diagnosis on their own. Loneliness has a detrimental impact on physical and psychological QoL [44,45,46,47,48,49,50,51]. This leads to a significant increase in the severity of depressive and anxiety symptoms. The results presented in this study show that lonely patients are more likely to have depression. In the available literature, loneliness is also considered a risk factor for depression [8,18,19]. However, it should be remembered that loneliness and depression interact with each other and have a synergistic effect on the deterioration of a patient’s well-being [18]. Polański et al. documented that mental disorders such as anxiety and depression may lead to worsening the functioning of the patient [5].

A number of factors have been found to be associated with loneliness. These include, among others, employment status, age, level of education and the type of coping strategy adopted. Economic inactivity and older age are strongly associated with increased levels of loneliness. The financial difficulties faced by those who do not have a job are also risk factors for malnutrition [50,52,53]. This study notes a higher risk of malnutrition as well as malnutrition in people suffering from loneliness. Similarly, in other studies, loneliness was considered a significant risk factor for malnutrition [49]. Lonely people are more likely to eat fewer meals, and they have difficulties in ensuring the right number of vegetables, fruits and proteins in their diet. Help from relatives in preparing meals and caring for a sick person gives better chances for a balanced diet and reduces the risk of malnutrition. It can also have an emotional impact, as the presence of a partner can encourage the patient to eat or even improve his appetite. Additionally, in a paper published by Chabowski et al., it is noted that patients at risk of malnutrition are more likely to suffer from severe depression and anxiety than patients whose nutritional status is normal [54]. Patients with a lower level of education may have difficulty finding information about nutrition after a cancer diagnosis and about coping with and understanding their illness.

Loneliness has a multidimensional impact on the patient because it is more difficult for lonely people to obtain information about nutrition, so they do not know how to take care of their diet. It also affects the patient emotionally and increases levels of anxiety and depression, so the patient has no appetite or motivation to properly take care of his nutritional status.

Driven by this lack of understanding and the feeling of guilt, patients often do not accept their illness. They feel angry and are thus more reluctant to follow their doctor’s advice on physical activity, a balanced diet and psychological support.

The constructive cancer coping model has a positive impact on illness acceptance and increases life satisfaction in patients [54]. In our study, lonely patients are less likely to choose constructive coping strategies such as positive redefinition and fighting spirit. Moreover, our analysis showed that a score of less than 41 for the use of constructive cancer coping strategies (Mini-MAC) is most strongly associated with being lonely. A positive coping strategy reduces the risk of social contact avoidance and thus lowers the risk of loneliness. As a result, patients are more willing to seek support from their family and friends as well as from a psychologist or a psychiatrist [55].

According to the authors, it is extremely important to teach the patient how to cope with cancer and always take into account that loneliness could limit the choice of a positive strategy. Knowing that constructive strategies have a positive impact on the quality of life and life satisfaction, health providers should strive to achieve them. It will allow them to achieve the greatest possible benefits for the patient and even avoid the effects of destructive strategies and loneliness that may intensify the negative coping strategies. The QoL of these patients can be improved through the provision of an appropriate level of support, balanced diet programmes aimed at preventing malnutrition, cancer adjustment and acceptance programmes, as well as through the prevention and treatment of anxiety and depression.

Achieving a better understanding of lung cancer and lung cancer stigma, including the impact of patient stigmatisation on loneliness, QoL and functioning, is important for healthcare professionals. It may enable the development of appropriate educational programmes and awareness-raising campaigns, which could be effective in improving the QoL of patients with lung cancer [45].

Authors suggest that there is a need to introduce screening for loneliness in cancer patients, along with tailored strategies for coping strategies and proper emotional support [36]. Clinicians should ensure support from patient’s family and their loved ones and take care to stop stigmatizing lung cancer patients. All the factors presented in this study affect the occurrence of loneliness, which affects the deterioration of the QoL, functioning in the five assessed dimensions (physical, psychological, cognitive, social and life roles), and even the level of nutrition. Therefore, we believe that the presented subject is an important issue to be used in everyday medical practice. Appropriate care for loneliness will ensure a fully holistic approach to cancer patients.

## 5. Conclusions

Loneliness is a significant factor affecting the functioning of patients with lung cancer. It increases the risk of anxiety and depression, reduces levels of illness acceptance, reduces levels of constructive coping and increases the risk of malnutrition.

Loneliness is an important clinical issue and should be taken into consideration when assessing the QoL and prognosis for the patient.

## Figures and Tables

**Table 1:** General characteristics of 310 patients divided into two groups based on their marital status.

Parameters	Lonely	Married or in a Relationship	*p*-Value
	N = 136	N = 174	
Age (years), mean ± SD	67.2 ± 8.4	61.1 ± 9.3	<0.001
Male sex, n (%)	75 (55.1)	102 (58.6)	0.564
Living in an urban area, n (%)	93 (68.4)	122 (70.1)	0.743
Employed, n (%)	14 (10.3)	72 (41.4)	<0.001
Education:			0.033
Primary, n (%)	20 (14.7)	14 (8.1)	
Vocational, n (%)	67 (49.3)	70 (40.2)	
Secondary, n (%)	36 (26.5)	67 (38.5)	
Tertiary, n (%)	13 (9.5)	23 (13.2)	
Type of tumor: SCLC, n (%)	29 (21.3)	47 (27.0)	0.242
TNM *:			
Tx, n (%)	3 (2.2)	2 (1.1)	0.082
T1, n (%)	25 (18.4)	26 (14.9)	
T2, n (%)	45 (33.1)	53 (30.5)	
T3, n (%)	22 (16.2)	16 (9.2)	
T4, n (%)	41 (30.1)	77 (44.3)	
Nx, n (%)	12 (8.8)	16 (9.2)	0.722
N0, n (%)	45 (33.1)	51 (29.3)	
N1, n (%)	28 (20.6)	32 (18.4)	
N2, n (%)	37 (27.2)	60 (34.5)	
N3, n (%)	14 (10.3)	15 (8.6)	
Mx, n (%)	15 (11.1)	26 (14.9)	0.686
M0, n (%)	82 (60.7)	95 (54.6)	
M1, n (%)	35 (25.9)	49 (28.2)	
M2, n (%)	3 (2.2)	4 (2.3)	
Number of hospital stays, Me (Q1–Q3)	1 (0–1.5)	1 (0–2)	0.096
Tumor metastases, n (%)	56 (41.2)	73 (42.0)	0.890
Bone metastases, n (%)	11 (8.1)	13 (7.5)	0.840
Brain metastases, n (%)	9 (6.6)	15 (8.6)	0.659
Liver metastases, n (%)	19 (14.0)	24 (13.8)	0.964
Adrenal gland metastases, n (%)	17 (12.5)	21 (12.1)	0.909
Spread of cancer to many organs through the bloodstream, n (%)	15 (11.0)	13 (7.5)	0.278

* TNM—The TNM classification of malignant tumors, Tx—tumor cannot be assessed; T1–T4—size and/or extension of the primary tumor; Nx—lymph nodes cannot be assessed; N0—no regional lymph nodes metastasis; N1—regional lymph node metastasis present, at some sites, tumor spread to closest or small number of regional lymph nodes; N2—tumor spread to an extent between N1 and N3; N3—tumor spread to more distant or numerous regional lymph nodes, Mx-cancers that cannot be evaluated for distant metastasis, M0—no distant metastasis, M1,M2—metastasis to distant organs beyond regional lymph nodes.

**Table 2:** Clinical characteristics of 310 patients divided into two groups based on their marital status.

Parameters	Lonely	Married or in a Relationship	*p*-Value
Surgery, n (%)	68 (50.0)	84 (48.3)	0.763
Symptoms, n (%)	101 (98.1)	144 (93.5)	0.131
Chronic cough, n (%)	88 (64.7)	109 (62.6)	0.708
Dyspnoea, n (%)	84 (61.8)	93 (53.4)	0.142
Pain in the chest, n (%)	47 (34.6)	72 (41.4)	0.220
Haemoptysis, n (%)	33 (24.3)	40 (23.0)	0.793
Recurring infections, n (%)	37 (27.2)	39 (22.4)	0.330
Superior vena cava syndrome, n (%)	3 (2.2)	7 (4.0)	0.522
Heart arrhythmia, n (%)	17 (12.5)	21 (12.1)	0.952
Hoarseness, n (%)	30 (22.1)	31 (17.8)	0.351
Number of comorbidities, Me (Q1–Q3)	1 (1–2)	1 (0–2)	0.016
Smoker, n (%)	54 (39.7)	78 (44.8)	0.365
FEV1/FVC (%), Me (Q1–Q3)	70.0 (63–75)	71.1 (66–75)	0.354
Degree of efficiency:			0.019
0	15 (11.0)	35 (20.1)	
1	51 (37.5)	78 (44.8)	
2	59 (43.4)	46 (26.4)	
3	8 (5.9)	9 (5.2)	
4	3 (2.2)	6 (3.5)	
Degree of obstruction, n (%)			0.487
Low, n (%)	13 (9.6)	12 (6.9)	
Moderate, n (%)	114 (83.8)	154 (88.5)	
Severe, n (%)	9 (6.6)	8 (4.6)	
Nutritional status:			0.275
Satisfactory nutritional status, n (%)	48 (35.3)	77 (44.3)	
Risk of malnutrition, n (%)	61 (44.9)	66 (37.9)	
Malnutrition, n (%)	27 (19.9)	31 (17.8)	
EORTIC QoL, Me (Q1–Q3)	68.9 (60.1–83.2)	71.2 (61.8–84.6)	0.748
EORTIC Func. SC, Me (Q1–Q3)	60.7 (46.7–80.3)	64.5 (48.7–87.3)	0.422
EORTIC Symp. 30SC, Me (Q1–Q3)	66.0 (53.7–84.0)	66.4 (53.7–82.7)	0.958
EORTIC Symp. 13SC, Me (Q1–Q3)	76.8 (69.7–86.9)	78.8 (68.7–88.9)	0.865
Mini-MAC Anxious Preoccupation	19.5 (17–23)	22 (17–24)	0.045
Mini-MAC Fighting Spirit	19 (17–21.5)	21 (19–23)	<0.001
Mini-MAC Helplessness-Hopelessness	16 (14–19)	15 (12–19)	0.051
Mini-MAC Positive Redefinition	19 (18–21.5)	21 (18–23)	0.001
Constructive cancer coping style	39 (34–42.5)	42 (38–45)	<0.001
Destructive cancer coping style	36 (32–40)	37 (32–41)	0.519
AIS (score)	22 (16.5–30)	25 (18–34)	0.017
VAS	5 (3–6)	5 (3–6)	0.975
HADS-Anxiety	19 (17–21)	19 (16–21)	0.102
HADS-Depression	18 (17–19)	18 (16–19)	0.034

**Table 3:** Number (percentage) of patients in groups differing in marital status and the results of the questionnaires used, the results of independence tests and the values of odds ratios and their 95% confidence intervals.

Marital Status	Evaluation of Mental Adjustment to Cancer (Mini-MAC Scale)				*p*	OR (95% CI)
	Mini-MAC AP < 22		Mini-MAC AP ≥ 22			
Lonely	85	50.9%	51	35.7%	0.008	1.87 (1.18–2.95)
Married or in a relationship	82	49.1%	92	64.3%		1.00 (ref.)
	Mini-MAC FS < 21		Mini-MAC FS ≥ 21			
Lonely	83	53.5%	53	34.2%	<0.001	2.22 (1.40–3.51)
Married or in a relationship	72	46.5%	102	65.8%		1.00 (ref.)
	Mini-MAC PR < 20		Mini-MAC PR ≥ 20			
Lonely	69	55.2%	67	36.2%	0.001	2.17 (1.37–3.45)
Married or in a relationship	56	44.8%	118	63.8%		1.00 (ref.)
	Mini-MAC CS < 41		Mini-MAC CS ≥ 41			
Lonely	86	55.5%	50	32.3%	<0.001	2.62 (1.65–4.16)
Married or in a relationship	69	44.5%	105	67.7%		1.00 (ref.)
	Acceptance of illness (AIS)					
	AIS < 21		AIS ≥ 21			
Lonely	61	53.5%	75	38.3%	0.013	1.86 (1.16–2.96)
Married or in a relationship	53	46.5%	121	61.7%		1.00 (ref.)
	Depressive disorders (HADS)					
	Depression ≥ 17		Depression < 17			
Lonely	116	48.1%	20	29.0%	0.006	2.27 (1.28–4.05)
Married or in a relationship	125	51.9%	49	71.0%		1.00 (ref.)

**Table 4:** Results of univariate and multivariate logistic regression analysis.

Loneliness-Related Risk Factors	Univariate Analysis		Multivariate Analysis		
	*b*	*p*	*beta*	*p*	OR [95% CI]
No employment	1.817	<0.001	1.542	<0.001	4.67 [2.14–10.2]
Age ≥ 61 years	1.350	<0.001	0.631	0.062	1.88 [0.97–3.65]
Mini-MAC CS score < 41	0.962	<0.001	0.159	0.753	1.17 [0.43–3.16]
Mini-MAC PR score < 20	0.775	0.001	0.653	0.082	1.92 [0.92–4.01]
Mini-MAC FS score < 21	0.797	0.001	0.045	0.915	1.05 [0.45–2.41]
Mini-MAC AP score < 22	0.626	0.008	0.198	0.516	1.22 [0.67–2.22]
Degree of efficiency: ≥2°	0.675	0.004	0.405	0.172	1.50 [0.84–2.68]
Cancer stage < T4	0.609	0.012	0.898	0.002	2.45 [1.39–4.32]
Two or more comorbidities	0.506	0.034	0.375	0.202	1.45 [0.82–2.59]
HADS Depression score ≥ 10	0.821	0.006	0.565	0.095	1.76 [0.91–3.42]
AIS score < 21	0.619	0.010	0.701	0.028	2.02 [1.08–3.77]

*b*—logistic regression coefficient, *beta*—standardised logistic regression coefficient, OR—odds ratio.

## Data Availability

Not applicable.
